# Genomic epidemiology of superspreading events in Austria reveals mutational dynamics and transmission properties of SARS-CoV-2

**DOI:** 10.1126/scitranslmed.abe2555

**Published:** 2020-11-23

**Authors:** Alexandra Popa, Jakob-Wendelin Genger, Michael D. Nicholson, Thomas Penz, Daniela Schmid, Stephan W. Aberle, Benedikt Agerer, Alexander Lercher, Lukas Endler, Henrique Colaço, Mark Smyth, Michael Schuster, Miguel L. Grau, Francisco Martínez-Jiménez, Oriol Pich, Wegene Borena, Erich Pawelka, Zsofia Keszei, Martin Senekowitsch, Jan Laine, Judith H. Aberle, Monika Redlberger-Fritz, Mario Karolyi, Alexander Zoufaly, Sabine Maritschnik, Martin Borkovec, Peter Hufnagl, Manfred Nairz, Günter Weiss, Michael T. Wolfinger, Dorothee von Laer, Giulio Superti-Furga, Nuria Lopez-Bigas, Elisabeth Puchhammer-Stöckl, Franz Allerberger, Franziska Michor, Christoph Bock, Andreas Bergthaler

**Affiliations:** 1 CeMM Research Center for Molecular Medicine of the Austrian Academy of Sciences, 1090 Vienna, Austria.; 2 Department of Data Sciences, Dana-Farber Cancer Institute, Boston, MA, USA.; 3 Department of Biostatistics, Harvard T.H. Chan School of Public Health, Boston, MA, USA.; 4 Department of Stem Cell and Regenerative Biology, Harvard University, Cambridge, MA 02138, USA.; 5 Austrian Agency for Health and Food Safety (AGES), 1220 Vienna, Austria.; 6 Center for Virology, Medical University of Vienna, 1090 Vienna, Austria.; 7 Bioinformatics and Biostatistics Platform, Department of Biomedical Sciences, University of Veterinary Medicine, 1210 Vienna, Austria.; 8 Institute for Research in Biomedicine (IRB), 08028 Barcelona, Spain.; 9 Institute of Virology, Medical University Innsbruck, 6020 Innsbruck, Austria.; 10 Department of Medicine IV, Kaiser Franz Josef Hospital, 1100 Vienna, Austria.; 11 Department of Internal Medicine II, Medical University of Innsbruck, 6020 Innsbruck, Austria.; 12 Department of Theoretical Chemistry, University of Vienna, 1090 Vienna, Austria.; 13 Research Group Bioinformatics and Computational Biology, Faculty of Computer Science, University of Vienna, 1090 Vienna, Austria.; 14 Center for Physiology and Pharmacology, Medical University of Vienna, 1090 Vienna, Austria.; 15 Institució Catalana de Recerca i Estudis Avançats (ICREA), Barcelona, Spain.; 16 Broad Institute of MIT and Harvard, Cambridge, MA, USA.; 17 Ludwig Center at Harvard, Boston, MA, USA.; 18 Center for Cancer Evolution, Dana-Farber Cancer Institute, Boston, MA, USA.; 19 Department of Laboratory Medicine, Medical University of Vienna, 1090 Vienna, Austria.

## Abstract

Superspreading events shaped the coronavirus disease 2019 (COVID-19) pandemic, and their rapid identification and containment are essential for disease control. Here, we provide a national-scale analysis of severe acute respiratory syndrome coronavirus 2 (SARS-CoV-2) superspreading during the first wave of infections in Austria, a country that played a major role in initial virus transmissions in Europe. Capitalizing on Austria’s well-developed epidemiological surveillance system, we identified major SARS-CoV-2 clusters during the first wave of infections and performed deep whole-genome sequencing of more than 500 virus samples. Phylogenetic-epidemiological analysis enabled the reconstruction of superspreading events and charts a map of tourism-related viral spread originating from Austria in spring 2020. Moreover, we exploited epidemiologically well-defined clusters to quantify SARS-CoV-2 mutational dynamics, including the observation of low-frequency mutations that progressed to fixation within the infection chain. Time-resolved virus sequencing unveiled viral mutation dynamics within individuals with COVID-19, and epidemiologically validated infector-infectee pairs enabled us to determine an average transmission bottleneck size of 10^3^ SARS-CoV-2 particles. In conclusion, this study illustrates the power of combining epidemiological analysis with deep viral genome sequencing to unravel the spread of SARS-CoV-2 and to gain fundamental insights into mutational dynamics and transmission properties.

## Tracking and tracing SARS-CoV-2 mutations

Austria was an early hotspot of SARS-CoV-2 transmission due to winter tourism. By integrating viral genomic and phylogenetic analyses with time-resolved contact tracing data, Popa *et al.* examined the fine-scale dynamics of viral spread within and from Austria in the spring of 2020. Epidemiologically defined phylogenetic clusters and viral mutational profiles provided evidence of the ongoing fixation of two viral alleles within transmission chains and enabled estimation of the SARS-CoV-2 bottleneck size. This study provides an epidemiologically contextualized, high-resolution picture of SARS-CoV-2 mutational dynamics in an early international transmission hub.

## Teaser

Epidemiological and genomic analyses uncover viral mutational dynamics and transmission bottleneck size during the early COVID-19 pandemic in Austria.

## INTRODUCTION

The severe acute respiratory syndrome coronavirus 2 (SARS-CoV-2) pandemic has already infected more than 20 million people in 188 countries, causing 737,285 deaths globally as of 11 August 2020 and extraordinary disruptions to daily life and national economies (*1*, *2*). The international research community rapidly defined pathophysiological characteristics of the coronavirus disease 2019 (COVID-19), established diagnostic tools, assessed immunological responses, and identified risk factors for a severe disease course (*3*–*6*). Clustered outbreaks and superspreading events of the SARS-CoV-2 pose a particular challenge to pandemic control (*7*–*10*). However, we still know comparatively little about fundamental properties of SARS-CoV-2 genome evolution and transmission dynamics within the human population.

Acquired fixed mutations enable phylogenetic analyses and have already led to insights into the origins and routes of SARS-CoV-2 spread (*11*–*14*). Conversely, low-frequency mutations and their changes over time within individual patients can provide insights into the dynamics of intrahost evolution. The resulting intrahost viral populations represent groups of variants with different frequencies, whose genetic diversity contributes to fundamental properties of infection and pathogenesis (*15*, *16*).

Austria is located in the center of Europe and has a population of 8.8 million. It operates a highly developed health care system, which includes a national epidemiological surveillance program. As of 7 August 2020, contact tracing had been performed for all 21,821 reported SARS-CoV-2–positive cases. Out of these, 10,385 cases were linked to epidemiological clusters, whereas no infection chains were identified for the remaining cases (*17*). Linked to Austria’s prominent role in international winter tourism, the country emerged as a potential superspreading transmission hub across the European continent in early 2020. During the first phase of the pandemic in Europe (February to May 2020), winter tourism–associated spread of SARS-CoV-2 from Austria may have been responsible for up to half of the imported cases in Denmark and Norway and a considerable share of imported cases in several other countries including Iceland and Germany (*11*, *18*, *19*).

In this study, we reconstructed major SARS-CoV-2 infection clusters in Austria and analyzed their role in international virus spread by combining phylogenetic and epidemiological analyses. Moreover, we analyzed our deep viral genome sequencing data from epidemiologically identified transmission chains and family clusters using biomathematical models, to infer genetic bottlenecks and the mutation dynamics of SARS-CoV-2 genome evolution. Our results provide fully integrated genetic and epidemiological evidence for continental spread of SARS-CoV-2 from Austria and establish fundamental transmission properties in the human population.

## RESULTS

### Genomic epidemiology reconstruction of SARS-CoV-2 infection clusters in Austria

**Fig. 1:**
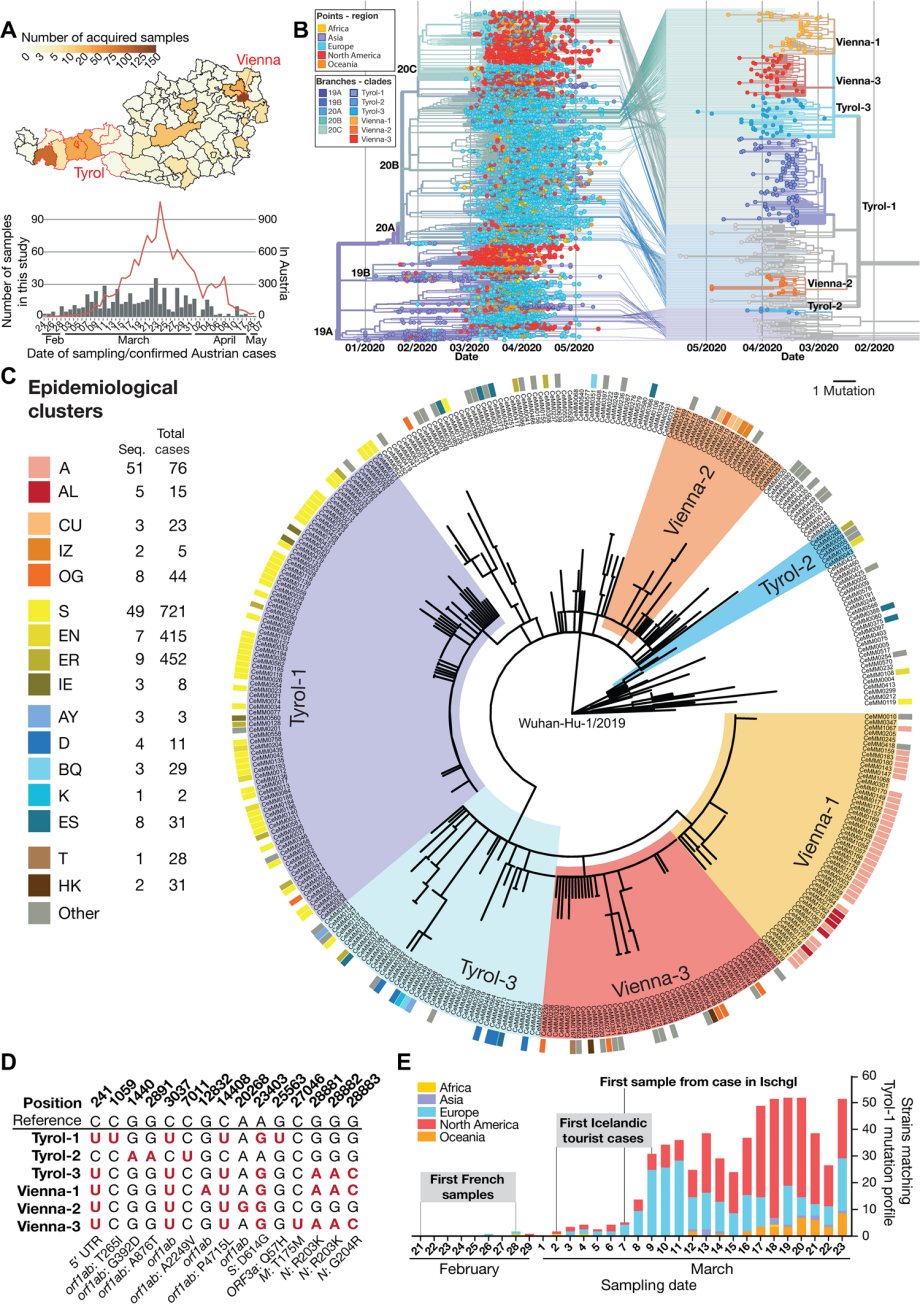
Phylogenetic-epidemiological reconstruction of SARS-CoV-2 infection clusters in Austria. (**A**) Number of acquired samples per district in Austria (top) and sampling dates of samples that underwent viral genome sequencing in this study (bottom), plotted in the context of all confirmed cases (red line) in Austria. (**B**) Connection of Austrian strains to global clades of SARS-CoV-2. Points indicate the regional origin of a strain in the time-resolved phylogenetic tree from 7666 randomly subsampled sequences obtained from GISAID including 345 Austrian strains sequenced in this study (left). Lines from global phylogenetic tree (left) to phylogenetic tree of all Austrian strains obtained in this study (right) indicate the phylogenetic relation and Nextstrain clade assignment of Austrian strains. Color schemes of branches represent Nextstrain clade assignment (left) or phylogenetic clusters of Austrian strains (right). (**C**) Phylogenetic tree of SARS-CoV-2 strains from Austrian patients with COVID-19 sequenced in this study. Phylogenetic clusters were identified on the basis of characteristic mutation profiles in viral genome sequences of SARS-CoV-2–positive cases in Austria. Cluster names indicate the most abundant location of patients based on epidemiological data. The circular color code indicates the epidemiological cluster assigned to patients based on contact tracing. (**D**) Mutation profiles of phylogenetic clusters identified in this study. Positions with characteristic mutations compared to reference sequence “Wuhan-Hu-1” (GenBank: MN908947.3) are highlighted in red. Details regarding the affected genes or genomic regions and the respective codon and amino acid change are given below the table. (**E**) Timeline of the emergence of strains matching the mutation profile of the Tyrol-1 cluster in the global phylogenetic analysis by geographical distribution with additional information from European phylogenetic reconstruction.

We selected and analyzed SARS-CoV-2 virus samples from geographical locations across Austria, with a focus on the provinces of Tyrol and Vienna, given that these two regions were initial drivers of the pandemic in Austria (fig. S1A) (*17*). We sequenced 572 SARS-CoV-2 RNA samples from 449 unique SARS-CoV-2 cases spanning a time frame between 24 February and 7 May. This captured both the onset and the peak of the initial COVID-19 outbreak in Austria (Fig. 1A). The selected samples covered multiple epidemiological and clinical parameters including age, sex, and viral load (fig. S1, B and C). Samples from both swabs (nasal and oropharyngeal) and secretions (tracheal and bronchial) were included (fig. S1D) to investigate the evolutionary dynamics not only within the population but also within individuals.

Of the 572 samples, 427 passed our sequencing quality controls (>96% genome coverage, >80% aligned viral reads, and ≤1500 uncalled nucleotides in the consensus sequences), and after the removal of cell culture samples, 420 samples were considered for low-frequency analysis. Of the 420 samples, 345 corresponded to unique SARS-CoV-2 cases and were further integrated in our phylogenetic analyses, as they corresponded to unique patient identifiers with complete sample annotation at the time of the analysis (fig. S1E). For these 345 samples, we assembled SARS-CoV-2 genome sequences, constructed phylogenies, and identified low-frequency mutations based on high-quality sequencing results with >5 million reads per sample and >80% of mapped viral reads (fig. S2, A and B).

To obtain robust quantifications of minor variants in all 420 samples, we validated our sample processing workflow and pipeline with additional experimental controls including synthetic SARS-CoV-2 genome titrations, technical replicates for sample preparation and sequencing runs, and dilution experiments (data file S1). Matched controls were highly consistent with each other, indicative of excellent assay performance and a highly reproducible analysis pipeline (fig. S2, C to F). For an alternative allele frequency of 0.01, we obtained an average accuracy of 90.92% (ranging from 68 to 97%). In addition, the shared percentage of detected variants between control pairs ranged from 50 to 90.97% for a cutoff of 0.02 of the allele frequencies. The high specificity of detection even at low frequencies, as well as the large overlap of detected variants, supported the choice of a 0.02 frequency cutoff for calling high-confidence variants (data file S1).

To investigate the link between local outbreaks in Austria and the global pandemic, we performed phylogenetic analysis of 345 SARS-CoV-2 genomes from Austrian cases and 7666 global genomes from the GISAID (Global Initiative on Sharing All Influenza Data) database (data file S2). Similar mutation profiles, together with information of geographical proximity of the samples and time of infection, are strong indicators of possible transmission links. Therefore, groups of virus sequences were annotated as phylogenetic clusters when they all shared a homogeneous mutation pattern and originated from the same geographical location and time period. Among the distinct phylogenetic clusters identified, six could be linked to specific geographic locations of the probable region of infection (Fig. 1B). Three of these six clusters comprised samples with a geographical location mainly in the Tyrol region (hereafter named Tyrol-1, Tyrol-2, and Tyrol-3), whereas the other three originated in Vienna (hereafter named Vienna-1, Vienna-2, and Vienna-3). These clusters are related to the global clades 19A, 20A, 20B, and 20C of the widely used Nextstrain classification (fig. S3A).

Independently, contact tracing surveillance assigns SARS-CoV-2 cases to epidemiological clusters based on the identification of transmission lines. In Austria, an extensive centralized tracing program was implemented during the COVID-19 outbreak. This program facilitated grouping of positive cases with a common exposure history and a comparable time frame of infection into epidemiological clusters. Integration of the phylogenetic analysis of Austrian SARS-CoV-2 sequences with epidemiological data resulted in strong overlap of these two lines of evidence, with 199 of the 345 sequences (65%) assigned to epidemiological clusters (data file S3). All sequenced samples from epidemiological cluster A mapped to the relatively homogeneous phylogenetic cluster Vienna-1 (Fig. 1C) with an index patient who had returned from Italy.

Our largest phylogenetic cluster, Tyrol-1 (fig. S3B), contained samples originating mainly from Austria’s Tyrol region (73 of 90 samples) and overlapped with epidemiological cluster S (44 of 53 epidemiologically annotated samples). This phylogenetic cluster included resident and travel-associated cases to the ski resort Ischgl or the related valley Paznaun (Fig. 1C). Although different SARS-CoV-2 strains circulated in the region of Tyrol, these data suggest that epidemiological cluster S originated from a single strain with a characteristic mutation profile leading to a large outbreak in this region. To elucidate the possible origin of the SARS-CoV-2 strain giving rise to this cluster, we searched for sequences matching the viral mutation profile among global SARS-CoV-2 sequences (Fig. 1, D and E). Using phylogenetic analysis, we found that the mutation profile defining the Tyrol-1 cluster matched the definition of the global clade 20C of the Nextstrain classification (fig. S3C). This clade is predominantly populated by strains from North America.

To reveal possible transmission lines specifically between European countries in February and March 2020, we performed phylogenetic analysis using all 7731 European high-quality SARS-CoV-2 sequences sampled before 31 March that were available in the GISAID database (data file S2). Using this approach, we identified several samples matching the Tyrol-1 cluster mutation profile from a local outbreak in the region Hauts-de-France in the last week of February (*20*). Introduction of this SARS-CoV-2 strain to Iceland by tourists with a travel history to Austria was reported as early as 2 March (Fig. 1E and fig. S3C) (*11*), indicating that viruses with this mutational profile were already present in Ischgl in the last week of February. These findings suggest that the emergence of cluster Tyrol-1 coincided with the local outbreak in France and with the early stages of the severe outbreak in northern Italy (*21*). The viral genomes observed in the Tyrol-1 cluster were closely related to those observed among the Icelandic cases with a travel history to Austria (fig. S3, D and E) (*11*). Vice versa, many of the Icelandic strains with a Tyrol-1 mutation profile had reported an Austrian or Icelandic exposure history (fig. S3F). Together, these observations and epidemiological evidence support the notion that the SARS-CoV-2 outbreak in Austria propagated to Iceland. Moreover, the emergence of these strains coincided with the emergence of the global clade 20C. One week after the occurrence of SARS-CoV-2 strains with this mutation profile in France and Ischgl, an increasing number of related strains based on the same mutation profile could be found across continents (Fig. 1E), for example, in New York City (*12*). As a popular skiing destination attracting thousands of international tourists, Ischgl may have played a critical role as transmission hub for the spread of clade 20C in Europe and beyond (fig. S3, G and H) (*12*). However, because of the lack of global epidemiological surveillance programs, it is rarely possible to infer direct transmission lines between countries.

Our results integrating epidemiological and sequencing data emphasize that phylogenetic analyses of SARS-CoV-2 sequences empower robust tracing from interindividual to local and international spreading events (*12*). Both clusters Tyrol-1 and Vienna-1 originated from crowded indoor events (an Apré Ski bar and a sports class, respectively), which are now appreciated as high-risk situations for superspreading events.

### Dynamics of low-frequency and fixed mutations in clusters

**Fig. 2:**
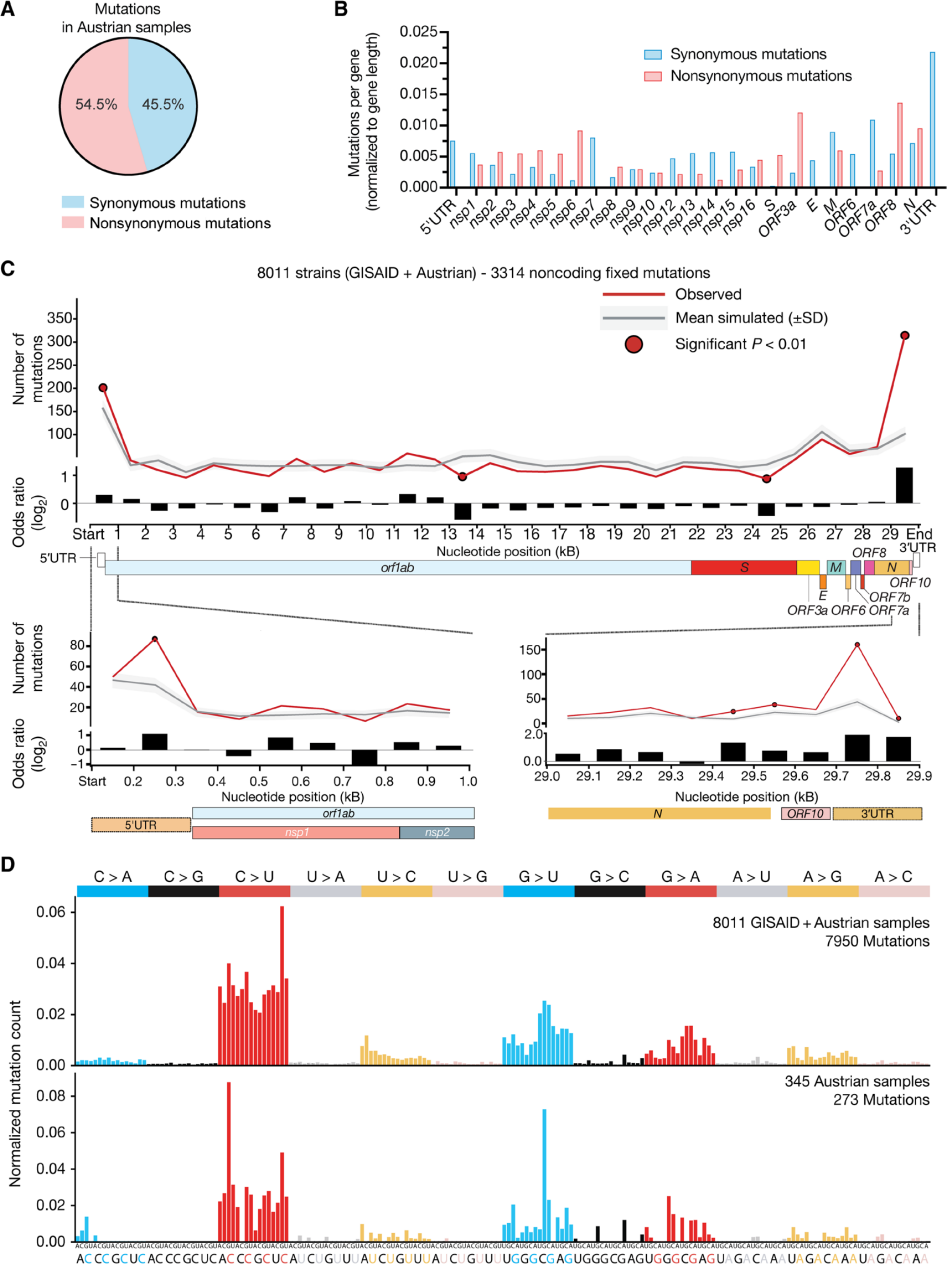
Mutational analysis of fixed mutations in SARS-CoV-2 sequences. (**A**) Ratio of nonsynonymous to synonymous mutations in unique mutations identified in Austrian SARS-CoV-2 sequences. (**B**) Frequencies of synonymous and nonsynonymous mutations per gene or genomic region normalized to length of the respective gene, genomic region, or gene product (*nsp1-16*). (**C**) Mutational spectra panel. Mutational profile of interhost mutations. Relative probability of each trinucleotide change for mutations across SARS-CoV-2 sequences in 7666 global sequences obtained from GISAID samples plus 345 Austrian samples (top) or 345 SARS-CoV-2 sequences from Austrian patients with COVID-19 (bottom). (**D**) Mutation rate distribution along the SARS-CoV-2 genome. Top: A 1-kb window comparison of the observed number of synonymous mutations across the global subsample of 8011 SARS-CoV-2 sequences from GISAID compared with the expected distribution (based on 10^6^ randomizations) according to their trinucleotide context. The gray line indicates the mean number of simulated mutations in the window, the colored background represents the distribution of expected mutations (mean ± SD), and red dots indicate a significant difference (G-test goodness of fit *P* < 0.01). Odds ratio in log_2_ scale of the observed compared with the expected number of synonymous mutations across the thirty 1-kb windows of the SARS-CoV-2 genome. Bottom: A zoom-in into the mutation rate across the first (left) and last (right) 1-kb windows. The comparisons were performed using ten 100–base pair windows. Gene annotations for SARS-CoV-2 genome are given below the top panel.

Next, we sought to uncover the mutational dynamics of SARS-CoV-2 during its transmission through the human population. We investigated the mutation profiles of our samples in terms of both fixed mutations (that drive the phylogenetic analyses) and the pool of low-frequency variants of each one of our samples. More than half of the fixed mutations in the Austrian SARS-CoV-2 genomes were nonsynonymous (Fig. 2A), most frequently occurring in nonstructural protein 6 (*nsp6*), open reading frame 3a (*ORF3a*), and *ORF8* (Fig. 2, B and C). An analysis of mutational signatures in the 7666 global strains and the Austrian subset of SARS-CoV-2 isolates showed a heterogeneous mutational pattern dominated by C > U, G > U, and G > A substitutions (Fig. 2D).

**Fig. 3:**
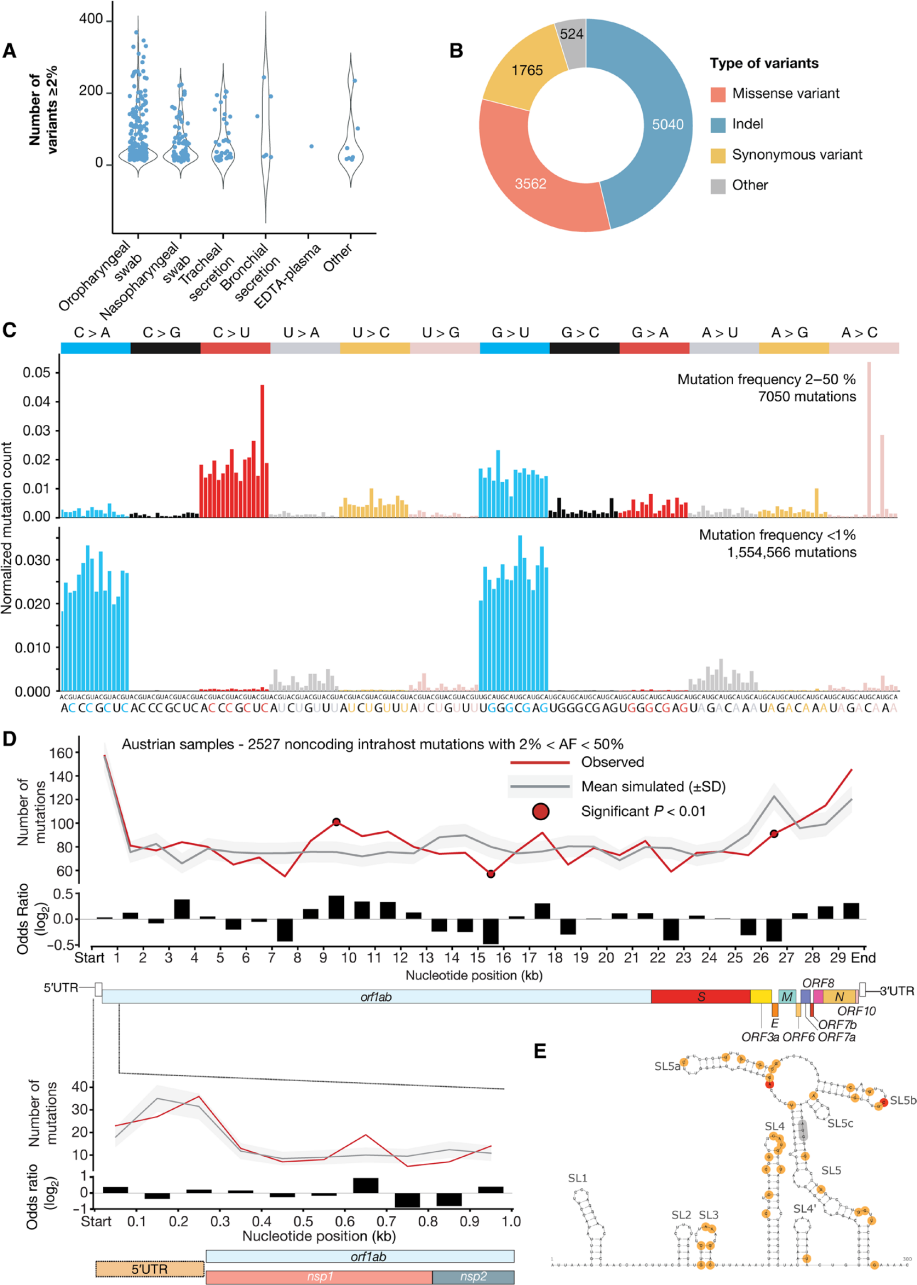
Analysis of low-frequency mutations. (**A**) Number of variants detected across different sample types. (**B**) Number of variants per variant class. (**C**) Mutational profile (relative probability of each trinucleotide) of 7050 intrahost mutations across Austrian samples (allele frequencies between 0.02 and 0.05) (top). Mutational profile (relative probability of each trinucleotide) of 1,554,566 intrahost mutations across Austrian samples (allele frequencies <0.01) (bottom). (**D**) Analysis of the mutation rate (analogous to the interhost mutation rate panel) across the SARS-CoV-2 genome using 2527 intrahost nonprotein affecting mutations with allele frequencies between 0.02 and 0.5. (**E**) RNA secondary structure prediction of the upstream 300 nucleotides of the SARS-CoV-2 reference genome (NC 045512.2), comprising the complete 5′ untranslated region (UTR) and parts of the nsp1 protein nucleotide sequence. The canonical AUG start codon is located in a stacked region of SL5 (highlighted in gray). Mutational hotspots observed in the Austrian SARS-CoV-2 samples are highlighted: Two fixed mutations at positions 187 and 241, respectively, are marked in red, and low-frequency variants with an abundance between 0.02 and 0.5 in individual samples are shown in orange. Insertion and deletion variants are not shown.

**Fig. 4:**
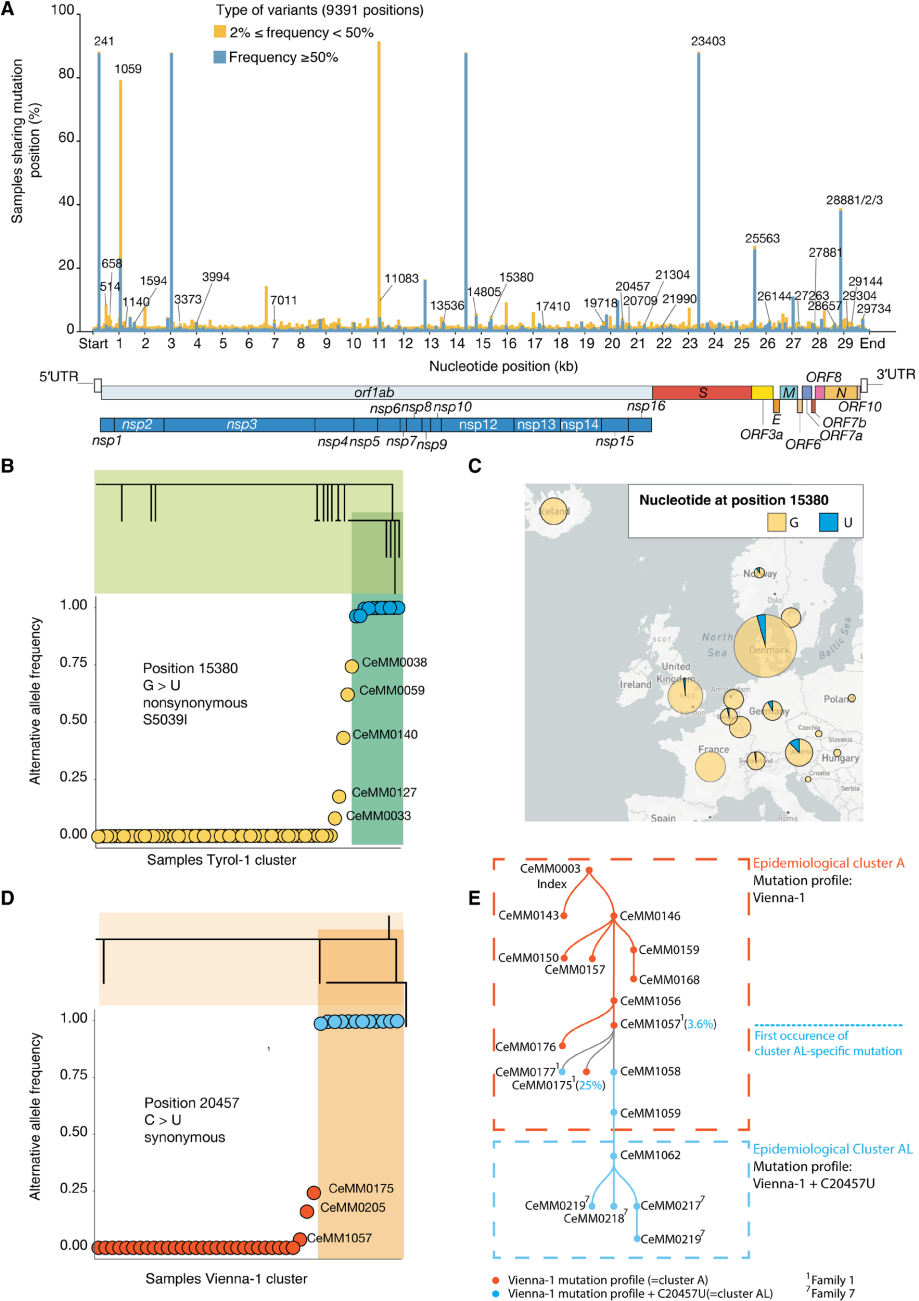
Dynamics of low-frequency and fixed mutations in superspreading clusters. (**A**) Percentage of samples sharing detected (≥0.02) mutations across genomic positions. For each of the 9391 positions harboring an alternative allele, the percentage of samples with high (≥0.50) or low [0.02, 0.50] frequency are reported in dark blue and orange, respectively. (**B**) Allele frequency of nonsynonymous mutation G > U at position 15,380 across samples in the phylogenetic cluster Tyrol-1. This variant has been observed both as low-frequency variant and as fixed mutation, the latter defining a phylogenetic subcluster (dark green). (**C**) Proportion of European samples with a reference (yellow) or alternative (blue) allele at position 15,380. (**D**) Allele frequency of synonymous mutation C > U at position 20,457 across samples of the Vienna-1 phylogenetic cluster. This variant is fixed and defines a phylogenetic subcluster (dark orange) as part of the broader Vienna-1 cluster. (**E**) Schematic representation of the transmission lines between epidemiological cluster A and cluster AL was reconstructed on the basis of results from deep viral sequencing and case interviews. The transmission scheme is overlaid with epidemiological clusters and family-related information.

We assessed the pool of variants for both low-frequency and fixed mutations (Fig. 3, A and B) and observed similar mutation patterns among these two sets of variants, which supports the accuracy of low-frequency mutation calling (Fig. 3, C and D). However, this pattern was lost for variants with an alternative frequency less than 0.01, which appear prone to false-positive variant calls. These results suggest that the same biological and evolutionary forces are at work for low-frequency and fixed mutations. Although the functional impact of variants across the genomes will need further research, we found that regions such as the 5′ untranslated region (5′UTR), which contains multiple stable RNA secondary structures, were subject to an increased mutation rate (Fig. 3D). Variants in the 5′UTR region are mainly localized along the stem-loop secondary structures (Fig. 3E). We found that 31% of the positions in the genome (9391 total positions) harbored variants (alternative allele frequency, ≥0.02) among the 420 sequenced strains from Austria and identified mutational hotspots for both high-frequency (≥0.5) and low-frequency (<0.5) mutations (Fig. 4A). Among these, 9034 positions exhibited only low-frequency mutations (<0.50), whereas four positions (241, 3037, 14,408, and 23,403) demonstrated fixation of the alternative allele in more than 50% of samples. We also identified 31 positions with alternative alleles being fixed in more than three samples and exhibiting a frequency <0.5 in at least two other samples (for example, 15,380 and 20,457).

On the basis of our phylogenetic analysis, we identified a subcluster inside the phylogenetic Tyrol-1 cluster that was defined by a fixed nonsynonymous G > U mutation at position 15,380 (Fig. 4B). This mutation was absent from all other Austrian cases but was detected at low and intermediary frequencies in other cases of the Tyrol-1 cluster. Around the time of emergence of this mutation, sequences sharing the same mutational profile (Tyrol-1 haplotype and G > U at position 15,380) appeared in other European countries including Denmark and Germany (Fig. 4C). Similarly, a synonymous fixed C > U mutation at position 20,457 defined a subcluster inside the phylogenetic Vienna-1 cluster (Fig. 4D). The cases from this subcluster intersected with members of two families (families 1 and 7) (Fig. 4E). Four members of family 1 tested positive for SARS-CoV-2 on 8 March and were epidemiologically assigned to cluster A. Yet, their viral sequences exhibited a wide range of C > U mutation frequencies at position 20,457 (0.00, 0.036, 0.24, and 1.00, respectively) (Fig. 4, D and E). Conversely, four members of family 7, who tested positive for SARS-CoV-2 between 16 and 22 March, were epidemiologically assigned to cluster AL and harbored viral genomes with a fixed U nucleotide at position 20,457 (Fig. 4, D and E).

**Fig. 5:**
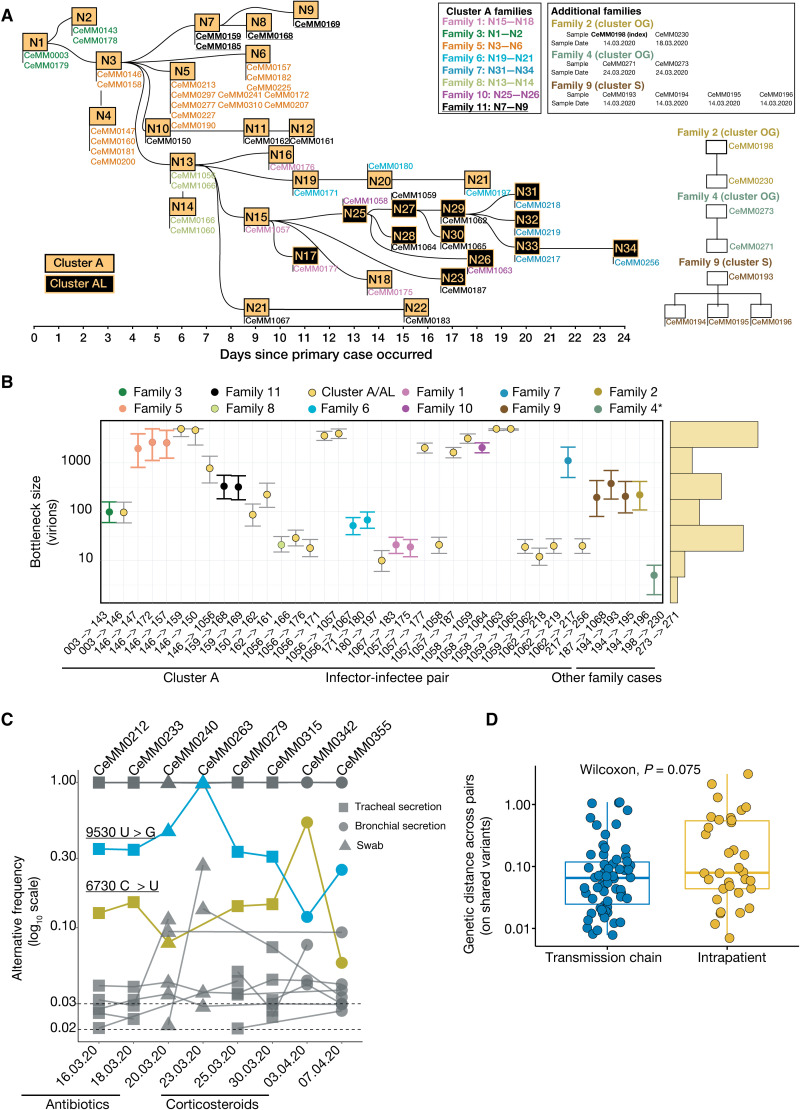
Impact of transmission bottlenecks and intrahost evolution on SARS-CoV-2 mutational dynamics. (**A**) Schematics of time-related patient interactions across epidemiological clusters A and AL. Each node represents a case, and links between the nodes are epidemiologically confirmed direct transmissions. Samples sequenced from the same individual are reported under the corresponding node. Cases corresponding to the same family are color coded accordingly. Additional families, unrelated to clusters A/AL, and their epidemiological transmission details are also reported. (**B**) Bottleneck size (number of virions that initiate the infection in an infectee) estimation across infector-infectee pairs based on the transmission network depicted in (A), ordered according to the timeline of cluster A for the respective pairs, and with a cutoff of [0.01, 0.95] for alternative allele frequency. For patients with multiple samples, the earliest sample was considered for bottleneck size inference. Centered dots are maximum likelihood estimates, with 95% confidence intervals. A star (*) for family 4 indicates that the transmission line was inferred as detailed in Materials and Methods. The histogram (yellow bars) of all the bottleneck values is provided on the right side of the graph. (**C**) Alternative allele frequency (*y* axis) of mutations across available time points (*x* axis) for patient 5. Only variants with frequencies ≥0.02 and shared between at least two time points are shown. Two mutations increasing in frequency are color coded. (**D**) Genetic distance values of mutation frequencies between infector-infectee pairs (A and B) (transmission chains) and intrapatient consecutive time points [(C) and fig. S5D]. Only variants detected in two same-patient samples were considered.

Through several telephone interviews, we followed up with the members of both families to reconstruct the timeline of the infection events (data file S4). Both grandparents of family 1 were exposed to infected case CeMM1056 (node N13; sampling date 3 March) during a recreational indoor event on 28 February and subsequently tested positive for SARS-CoV-2 (Figs. 4, D and E, and 5A). The woman, CeMM0176 (node N16; sampling date 8 March), did not present a mutation at position 20,457, whereas her husband, CeMM1057 (node N15; sampling date 6 March), had the U allele at this position with a frequency of 0.036. The chain of transmission continued in family 1 with the infection of the couple CeMM0175 (node N18; sampling date 8 March) and CeMM0177 (node N17; sampling date 8 March), who had the U mutation at frequencies of 0.25 and 1, respectively. All further transmissions from CeMM1057 (node N15) resulted in a fixed mutation at position 20,457. CeMM1058 (node N25; sampling date 8 March) was in contact with CeMM1057 on 2 March and attended a funeral on 5 March with CeMM1059 (node N27; sampling date 11 March). On March 8, multiple persons participated at a birthday party, which included case CeMM1059 together with CeMM1062 (node N29; sampling date 13 March). Case CeMM1062 was part of a choir with multiple members of family 7 [CeMM0218 (node N31), CeMM0219 (node N32), and CeMM0217 (node N33)] on 10 March (Figs. 4, D and E, and 5A). Given our phylogenetic analysis and epidemiological reconstruction of transmission chains, we thus provide strong evidence for the emergence of a fixed mutation within a family and its spreading across previously disconnected epidemiological clusters. Together, these results from two superspreading events (Tyrol-1 and Vienna-1) demonstrate the power of deep viral genome sequencing in combination with detailed epidemiological data for observing viral mutation on their way from emergence at low frequency to fixation.

### Impact of transmission bottlenecks and intrahost evolution on SARS-CoV-2 mutational dynamics

The emergence and potential fixation of mutations in the viral populations within a patient depend on interhost bottlenecks and intrahost evolutionary dynamics (*22*, *23*). An examination of the individual contributions of these forces requires pairs of samples from validated transmission events. For this purpose, we combined intrafamily cases, known epidemiological transmission chains, and subsequent telephone investigations to track the index cases as well as the context, date, and nature of each transmission event (Fig. 5A and data file S4) (*22*, *24*). Our set of SARS-CoV-2–positive cases comprised 39 epidemiologically confirmed infector-infectee pairs (Fig. 5A, fig. S4A, and data file S4).

One particularly well-defined network of SARS-CoV-2 transmission events linked cases from epidemiological cluster A and AL (Figs. 4E and 5A). The index case of cluster A is CeMM0003 (node N1), who contracted the virus during a visit to the north of Italy, further infecting his family members and, later, case CeMM0146 (node N3) during a dinner meeting (*17*). Multiple infections were linked to case CeMM0146 through an indoor sports activity. Among these cases was CeMM1056 (node N13), who further transmitted the virus to case CeMM1057 (node N15) as previously described for the 20,457 mutation linking cluster A and AL (Fig. 4E) (*17*). On the basis of these data, we investigated the transmission dynamics between known pairs of infectors and infectees by inferring the number of virions initiating the infection, also known as the genetic bottleneck size (*22*, *24*). The quality of the samples and the underlying low-frequency variants are critical for computing robust bottleneck sizes. In our data, samples with low Ct values (≤28) resulted in the detection of 38.6 variants (cutoff of 0.02) on average. Samples with high Ct values (>28) had on average 109.1 variants. The samples in the transmission chain were of high quality, with an average Ct value of 22.2, and only 9 of the 43 samples were higher than 28 (fig. S4A and data file S4).

Bottleneck size estimates were calculated by comparing the frequency of detected variants in each transmission pair (fig. S4, B to E). In particular, we computed bottleneck size using the beta-binomial method (*24*) and on three sets of alternative frequency cutoffs: [0.01, 0.95], [0.02, 0.95], and [0.03, 0.95] (fig. S4F and data file S4). Although the absolute values of the estimates were influenced by these cutoffs, their underlying average bottleneck sizes were comparable: 1227.59 (25 and 75% quartile: 21 to 2053.5; SD, 1692.235), 1110.513 (25 and 75% quartile: 2.5 to 2115; SD, 1661.183), and 1319.41 (25 and 75% quartile: 3.5 to 1763; SD, 1685.378) for the 0.01, 0.02, and 0.03 cutoffs, respectively (Fig. 5B and fig. S4G). In conclusion, taking advantage of a well-described and independently confirmed transmission network with 39 transmission events, we found that the number of viral particles transmitted from one individual to another that contributed productively to the infection was on average higher than 1000.

Last, we investigated the dynamics of intrahost evolution by using time-resolved viral sequences from 31 longitudinally sampled patients. These patients were subject to different medical treatments, and five of them succumbed to COVID-19–related complications (data file S5). To analyze intrahost viral dynamics, we focused on variants observed in at least two samples from the same patient. This approach resulted in a pool of high-confidence mutations (>0.02) with high coverage across same-patient samples (mean, 42,099 reads) (fig. S5A). Same-patient samples shared more variants than unrelated sample pairs (defined as non–same-patient, nor from the transmission chains) (fig. S5B). In addition, variants shared between samples from the same patient were unlikely to be found in unrelated samples (fig. S5C).

We observed diverse mutation patterns across individual patients and over time. Most patient samples showed a small number of stable low-frequency mutations (≥0.02 and ≤0.50), whereas cases CeMM0108, CeMM0172, CeMM0251, CeMM0269, CeMM0299, and CeMM0221 exhibited higher variability, including the fixation and loss of individual mutations (Fig. 5C and fig. S5D). The patient-specific dynamics of viral mutation frequencies may reflect the effect of host-intrinsic factors such as immune responses or the patients’ overall health, and extrinsic factors such as different treatment protocols. We also examined the genetic distance between samples obtained across infector-infectee pairs and serially acquired patient samples. However, the difference between increased genetic divergence of the virus within individual patients over the course of infection compared with interhost transmission was not significant (*P* = 0.075) (Fig. 5D).

## DISCUSSION

Unprecedented global research efforts are underway to counter the COVID-19 pandemic around the globe and its pervasive impact on health and socioeconomics. These efforts include the genetic characterization of SARS-CoV-2 to track viral spread and to investigate the viral genome as it undergoes changes in the human population. Here, we leveraged deep viral genome sequencing in combination with national-scale epidemiological workup to reconstruct Austrian SARS-CoV-2 clusters that played a substantial role in the international spread of the virus. Our study describes how emerging low-frequency mutations of SARS-CoV-2 became fixed in local clusters, followed by viral spread across countries, thus connecting viral mutational dynamics within individuals and across populations. Exploiting our well-defined epidemiological clusters, we determined the interhuman genetic bottleneck size for SARS-CoV-2—which is the number of virions that start the infection and produce progeny in the viral population—at around 10^3^. Our estimated bottlenecks are based on a substantial number of defined infector-infectee pairs and in agreement with recent studies implying larger bottleneck sizes for SARS-CoV-2 compared with estimates for the influenza A virus (*22*, *25*–*28*). These bottleneck sizes correlated inversely with higher mutation rates of influenza virus as compared with SARS-CoV-2.

In agreement with our experimentally determined bottleneck sizes, a recent preprint describing a dose-response modeling study estimated 3 × 10^2^ to 2 × 10^3^ SARS-CoV-2 virions necessary to initiate an infection (*29*). The dynamics of superspreading events seem to be driven by the number of interindividual contacts and the quantity of transmitted virus over time (*29*). Accordingly, our relatively large observed bottleneck size could be the result of patient exposure to high virus accumulations in shared and closed space and may have been influenced by a lack of protective measures in the early phase of the first COVID-19 wave in spring 2020. Although we inferred an average bottleneck size of 10^3^ viral particles on average, the broad range of these values indicates that lower numbers of transmitted particles may also lead to a successful infection.

Our sequencing approach resulted in high-confidence variant calling and robust genome-wide coverage; hence, it is unlikely that technical limitations constituted a major source of bias. However, estimates of viral bottleneck sizes are likely influenced by many parameters not covered in this study, including virus-specific differences and stochastic evolutionary processes (*28*). Successful viral transmission also depends on other factors including the rate of decay of viral particles, frequency of susceptible cells, the host immune response, and comorbidities (*22*, *30*). The cases we analyzed were subject to different clinical contexts and treatments as well as disease outcomes. To better understand the mechanisms at work during infection, future investigations will need to probe these factors in the context of viral intrahost diversity across body compartments and time (*31*–*34*).

This study underscores the value of combining epidemiological approaches with virus genome sequencing to provide critical information to help public health experts track pathogen spread. Our genomic epidemiology analysis enabled the retrospective identification of SARS-CoV-2 chains of transmission and international hotspots such as the phylogenetic cluster Tyrol-1 (*14*, *35*–*37*). We also found that the Tyrol clusters were heterogeneous with regard to the S protein D614G mutation, which has been reported to contribute to viral transmissibility and fitness (*38*–*41*). Moreover, our phylogenetic analysis of the Vienna-1 cluster demonstrated the practical utility of viral genome sequencing data for uncovering previously unknown links between epidemiological clusters. This result was subsequently confirmed by follow-up contact tracing. We presented this case as an example of how the integration of contact tracing and sequencing information supports tracking the emergence and development of clusters. This demonstrates that deep viral genome sequencing can contribute directly to public health efforts by enhancing epidemiological surveillance.

Since the onset of the SARS-CoV-2 outbreak, many pandemic containment strategies have been implemented across the world. Where effective, these measures led to the reduction in the number of positive cases and limited superspreading events such as those investigated in this study. We found that most of the investigated infections likely involved the effective transmission of at least 1000 viral particles between individuals, suggesting that social distancing and mask wearing may be effective even when they cannot prevent the spread of all viral particles. As a future perspective, our study supports the relevance of investigating viral genome evolution of SARS-CoV-2 to enable informed decision-making by public health authorities (*42*).

## MATERIALS AND METHODS

### Study design

The goal of this study was to analyze mutational patterns in the SARS-CoV-2 genome to infer transmission in the human population from interindividual to global scale. For this purpose, isolated viral RNA from 572 Austrian samples (February to May 2020) was processed for genome consensus sequence reconstruction and variant calling as approved by the ethics committee of the Medical University of Vienna. Additional analyses on subsets of samples consisted of the profiling of the mutational patterns across the genome and bottleneck size estimates based on transmission pairs. Data presented in this study are based on epidemiological and contact tracing data from the Austrian Department of Infection Epidemiology & Surveillance at the Austrian Agency for Health and Food Safety (AGES).

### Sample collection and processing

Patient samples were obtained from the Medical Universities of Vienna Institute of Virology, Medical University of Innsbruck Institute of Virology, Medical University of Innsbruck Department of Internal Medicine II, Central Institute for Medical-Chemical Laboratory Diagnostics Innsbruck, Klinikum Wels-Grieskirchen, and AGES. Samples were obtained from suspected or confirmed SARS-CoV-2 cases or contact persons of these. Sample types included oropharyngeal swabs, nasopharyngeal swabs, tracheal secretion, bronchial secretion, serum, plasma, and cell culture supernatants. RNA was extracted using the following commercially available kits by adhering to the manufacturers’ instructions: MagMax (Thermo Fisher Scientific), EasyMag (bioMérieux), AltoStar Purification Kit 1.5 (Altona Diagnostics), MagNA Pure LC 2.0 (Roche), MagNA Pure Compact (Roche), and QIAsymphony (Qiagen). Viral RNA was reverse transcribed with Superscript IV Reverse Transcriptase (Thermo Fisher Scientific). The resulting complementary DNA was used to amplify viral sequences with modified primer pools from the Artic Network initiative (*43*). Polymerase chain reactions were pooled and subjected to high-throughput sequencing.

### Sample sequencing

Amplicons were cleaned up with AMPure XP beads (Beckman Coulter) with a 1:1 ratio. Amplicon concentrations were quantified with the Qubit Fluorometric Quantitation system (Life Technologies), and the size distribution was assessed using the 2100 Bioanalyzer system (Agilent). Amplicon concentrations were normalized, and sequencing libraries were prepared using the NEBNext Ultra II DNA Library Prep Kit for Illumina (New England Biolabs) according to the manufacturer’s instructions. Library concentrations again were quantified with the Qubit Fluorometric Quantitation system (Life Technologies), and the size distribution was assessed using the 2100 Bioanalyzer system (Agilent). For sequencing, samples were pooled into equimolar amounts. Amplicon libraries were sequenced on the NovaSeq 6000 platform (Illumina) using S Prime (SP) flowcell with a read length of 2 × 250 base pairs in paired-end mode.

### Sequencing data processing and analysis

Following demultiplexing, fastq files containing the raw reads were inspected for quality criteria (base quality, N and GC content, sequence duplication, and overrepresented sequences) using FastQC (v.0.11.8) (*44*). Trimming of adapter sequences was performed with BBDUK from the BBtools suite (http://jgi.doe.gov/data-and-tools/bbtools). Overlapping read sequences within a pair were corrected for using BBMERGE function from BBTools. Read pairs were mapped on the combined Hg38 and SARS-CoV-2 genome (GenBank: MN908947.3, RefSeq: NC_045512.2) using the BWA-MEM software package with a minimal seed length of 17 (v0.7.17) (*45*). BWA-MEM accounts for mismatches, insertions, and deletions in the alignment score and the mapping quality. Only reads mapping uniquely to the SARS-CoV-2 viral genome were retained. Primer sequences were removed after mapping by masking with iVar (*46*). From the viral reads BAM (binary alignment map) file, the consensus FASTA file was generated using Samtools (v1.9) (*47*), mpileup, Bcftools (v 1.9) (*47*), and SEQTK (https://github.com/lh3/seqtk). For calling low-frequency variants, the viral read alignment file was realigned using the Viterbi method provided by LoFreq (v2.1.2) (*48*). After adding InDel qualities, low-frequency variants were called using LoFreq. Variant filtering was performed with LoFreq and Bcftools (v1.9) (*49*). Only variants with a minimum coverage of 75 reads, a minimum phred value of 90, and indels (insertions and deletions) with an HRUN of minimum 4 were considered. All analyses except for the control analysis in Fig. 3C were performed on variants with a minimum alternative frequency of 0.01. The cutoff for the alternative frequency mainly used in this study was set to 0.02, except for Fig. 5B. Annotations of the variants were performed with SnpEff (v4.3) (*50*) and SnpSift (v4.3) (*51*).

### Epidemiological analyses and identification of SARS-CoV-2 infection clusters

The investigation of transmission chains (contact tracing) was conducted by the Department of Infection Epidemiology & Surveillance at the AGES. Epidemiological clusters were defined as accumulations of cases within a certain time period in a defined region and with common source of exposure. The required information for cluster annotation and resolution in chains of transmission was collected during the official case contact tracing by the public health authorities, resulting in identification of the most likely source cases and successive cases of the index cases. Contact tracing was performed according to technical guidance relating to this measure produced by the European Centre for Disease Prevention and Control (ECDC) (*52*). For refinement and validation of contact tracing data for cluster A and cluster AL, we contacted 17 cases for 15-minute interviews. The interviews comprised 10 questions concerning the most likely source, time, place, and setting of transmission, contact persons, and the course of disease (start and end of symptoms, kind of symptoms, severity, and hospitalization).

### Phylogenetic analysis and inference of transmission lines

Phylogenetic analysis was conducted using the Augur package (version 7.0.2) (*53*). We compiled a randomly subsampled dataset of 7666 full-length viral genomes with high coverage (<1% Ns) that were available from GISAID (https://gisaid.org/, 2 June) and the 345 sequences obtained in this publication. GISAID sequences were filtered for entries from human hosts with complete sampling dates. Metadata information for patient age and sex was excluded from the analysis. Multiple sequence alignments were performed using mafft (*54*). A masking scheme for homoplasic and highly ambiguous sites was applied to avoid bias in the following phylogenetic analysis as discussed elsewhere (*55*). We reconstructed the phylogeny with the augur pipeline using IQ-TREE (*54*) and further processed the resulting trees with treetime to infer ancestral traits of the nodes (*56*). Phylogenetic trees were rooted with the genome of “Wuhan-Hu-1/2019.” The same workflow was repeated for phylogenetic reconstruction of all high-quality European strains before 31 March 2020 available in the GISAID database by 7 June 2020 (7731). Clade annotations for global trees were adapted from nextstrain.org (https://github.com/nextstrain/ncov/blob/master/defaults/clades.tsv; https://clades.nextstrain.org/); clusters of Austrian strains were identified on the basis of shared mutation profiles and patient location from epidemiological data.

### Bottleneck estimation

Our analysis to estimate the transmission bottleneck sizes for each infector-infectee pair was based on the beta-binomial method presented in (*24*). For a given variant present in the infector, this method assumes that the number of transmitted virions carrying the variant is binomially distributed with the bottleneck size as the number of trials and success probability as the variant frequency in the infector. Following transmission, the viral population during early infection is modeled as a linear birth-death process, implying that the proportion of the viral population descended from any virion in the bottleneck population is beta-distributed. Using this model for the change in variant frequencies between infector and infectee pairs and assuming independence of mutations lead to the likelihood model of (*24*). Maximum likelihood analysis then provides the bottleneck statistics. Error bars denote 95% confidence intervals, determined by a likelihood ratio test. This method was applied to variants in the following frequency ranges: [0.01, 0.95], [0.02, 0.95], and [0.03, 0.95]. Because of the high sequencing depth of our study, we used the approximate version of the beta-binomial method.

### Intrapatient time series analyses

Among our 420 high-quality SARS-CoV-2–positive samples, we had 31 unique cases with multiple time-point samplings (a total of 106 samples). Nineteen of 31 cases had only two samples per patient. For each of the 31 cases, we only considered variants with an alternative frequency greater than 0.02 and that were shared across at least two of the intrapatient samples. We retrieved the depth of coverage of the selected variants for each sample for each patient. To compare how many variants were shared intrapatient as opposed to unrelated samples, we first identified potentially unrelated cases by eliminating all samples from the same patient, as well as all the samples in the transmission chains in Fig. 5A, resulting in 281 samples hereafter termed “unrelated.” We then enumerated all 39,340 unordered pairs of the 281 unrelated samples. Only variants between 0.02 and 0.5 were considered. We computed the percentage of variants shared by each pair out of the total number detected across the two samples. We then compared the percentage of variant sharing between intrapatient and unrelated pairs of samples with a Wilcoxon test. To test how widely the intrapatient variants ([0.02, 0.5]; 173 positions) were detected in other samples, we examined how often they were detected in the pool of 218 unrelated samples.

### Genetic distance

For shared mutations with defined infector-to-infectee transmission, we determined those mutations present in both samples and calculated their absolute difference in frequency. Similarly, we performed the same computations between time consecutive pairs for serially sampled patients. If multiple samples were obtained on the same day, the sample with the lowest Ct value was considered. Note that the time-consecutive pairs had a differing number of days between samples. To these genetic distances obtained from the shared variants, we added the sum of the frequencies of the variants detected in only one of the pairs of shared samples; that is, we calculated the l1-norm of the variant frequencies. Statistical difference between the genetic distances from transmission pairs versus consecutive pairs from serially sampled patients was determined by a Wilcoxon (one-sided) rank sum test.

### Statistical methods

Control samples were compared with a linear regression method, and the corresponding *R*^2^ was reported. For mutational patterns analyses, a statistical test was devised to compare the deviation of the observed number of mutations from the expected distribution as detailed in Materials and Methods. The frequency of mutations in overlapping windows across the genome was statistically assessed with a log-likelihood test. For bottleneck size computations, a maximum likelihood approach was applied. The comparison of genetic diversity between groups was performed with a standard Wilcoxon test. Significance was inferred for *P* values ≤0.05.

## SUPPLEMENTARY MATERIALS

stm.sciencemag.org/cgi/content/full/scitranslmed.abe2555/DC1 Materials and Methods Fig. S1. Data overview. Fig. S2. Technical pipeline and controls. Fig. S3. Phylogenetic analysis of SARS-CoV-2 sequences from Austrian patients with COVID-19 in global context. Fig. S4. Bottleneck size estimations. Fig. S5. Viral intrahost diversity in individual patients. Data file S1. Sample and sequencing information of the 572 samples and controls. Data file S2. Acknowledgments for SARS-CoV-2 genome sequences derived from GISAID. Data file S3. Epidemiological clusters referred to in this study. Data file S4. Transmission chain and sample information for cluster A/cluster AL and family-related cases. Data file S5. Clinical information of patients with COVID-19 relating to Fig. 5 and fig S5. Reference (*57*)

## Appendix

View/request a protocol for this paper from *Bio-protocol*.
